# Development and validation of a nomogram to predict cervical lymph node metastasis in head and neck squamous cell carcinoma

**DOI:** 10.3389/fonc.2023.1174457

**Published:** 2024-01-04

**Authors:** Xiaohan Chen, Lu Zhang, Haijun Lu, Ye Tan, Bo Li

**Affiliations:** ^1^ Department of Radiation Oncology, Affiliated Hospital of Qingdao University, Qingdao, China; ^2^ Department of Oncology and Radiotherapy, Affiliated Hospital of Qingdao University, Qingdao, China

**Keywords:** head and neck squamous cell carcinoma (HNSCC), nomogram, pathologic, lymph node, image

## Abstract

**Background:**

Head and neck cancers are a heterogeneous, aggressive, and genetically complex collection of malignancies of the oral cavity, nasopharynx, oropharynx, hypopharynx, larynx, paranasal sinuses and salivary glands, which are difficult to treat. Regional lymph nodes metastasis is a significant poor prognosis factor for head and neck squamous cell carcinoma. Metastasis to the regional lymph nodes reduces the 5-year survival rate by 50% compared with that of patients with early-stage disease. Accurate evaluation of cervical lymph node is a vital component in the overall treatment plan for patients with squamous cell carcinoma of the head and neck. However, current models are struggle to accurately to predict cervical lymph node metastasis. Here, we analyzed the clinical, imaging, and pathological data of 272 patients with HNSCC confirmed by postoperative pathology and sought to develop and validate a nomogram for prediction of lymph node metastasis in patients with head and neck squamous cell carcinoma.

**Methods:**

We retrospectively analyzed the clinical, imaging, and pathological data of 272 patients with head and neck squamous cell carcinoma (HNSCC) confirmed by postoperative pathology at the Affiliated Hospital of Qingdao University from June 2017 to June 2021. Patients were randomly divided into the training and validation cohorts in a 3:1 ratio, and after screening risk factors by logistic regression, nomogram was developed for predicting lymph nodes metastasis, then the prediction model was verified by C-index, area under curve (AUC), and calibration curve.

**Results:**

Of the 272 patients, seven variables were screened to establish the predictive model, including the differentiation degree of the tumor [95% confidence interval(CI):1.224~6.735, *P*=0.015], long-to-short axis ratio of the lymph nodes (95%CI: 0.019~0.217, *P*<0.001), uneven/circular enhancement (95%CI: 1.476~16.715, *P*=0.010), aggregation of lymph nodes (95%CI:1.373~10.849, *P*=0.010), inhomogeneous echo (95%CI: 1.337~23.389, *P*=0.018), unclear/absent medulla of lymph nodes (95%CI: 2.514~43.989, *P*=0.001), and rich blood flow (95%CI: 1.952~85.632, *P*=0.008). The C-index was 0.910, areas under the curve of training cohort and verification cohort were 0.953 and 0.938 respectively, indicating the discriminative ability of this nomogram. The calibration curve showed a favorable compliance between the prediction of the model and actual observations. The clinical decision curve showed this model is clinically useful and had better discriminative ability between 0.25 and 0.9 for the probability of cervical LNs metastasis.

**Conclusions:**

We established a good prediction model for cervical lymph node metastasis in head and neck squamous cell carcinoma patients which can provide reference value and auxiliary diagnosis for clinicians in making neck management decisions of HNSCC patients.

## 1 Introduction

Lymph nodes(LNs) metastases is an important prognosis indicator for patients with head and neck squamous cell carcinoma(HNSCC) (1). According to the 2020 National Comprehensive Cancer Network guidelines, for HNSCC patients with LNs metastases, radical cervical LNs dissection and necessary postoperative chemoradiotherapy are required, while elective dissection or non-dissection of cervical LNs is more appropriate for patients without LNs metastases. Imaging is an effective tool for identifying lymph node metastases, but no single imaging technique can accurately diagnose, stage, and provide long-term monitoring of HNSCC. The combination of ultrasound and computed tomography(CT) may be helpful, but neither of them has 100% diagnostic sensitivity (2). Therefore, a comprehensive and systematic approach based on previous research is needed to maximize the benefit of preoperative imaging techniques. As a chart, nomogram could assess the incidence of clinical events quickly, visually, and accurately. Their validity has been demonstrated in assessing the probability of cervical LNs metastasis in some HNSCCs (including tongue, larynx, and hypopharynx) (3–5). However, they only evaluated the correlation between clinicopathological features and cervical LNs metastasis.

In this study, we constructed a novel predict model and validated it for predicting cervical LNs metastasis in patients with HNSCC based on the incorporated imaging features of cervical LNs and pathological features of primary tumor. Compared to other models, our study included more variables and exhibited superior discrimination capabilities. According to the risk stratification based on this nomogram, this prediction model can provide clinicians with aid in diagnosis, high-risk patients may profit from neck management, but low-risk patients may be conservative. We present this article in accordance with the TRIPOD reporting checklist.

## 2 Methods

### 2.1 Patient selection

This was a retrospective study compliant with the Declaration of Helsinki (as revised in 2013). All included patients gave their oral and written informed consent. This study was approved by the Institutional Review Board of the affiliated hospital of Qingdao University (No. QYFY WZLL 27167). We collected patients with HNSCC confirmed by postoperative pathology at the Affiliated Hospital of Qingdao University from June 2017 to June 2021. After acquiring complete information, following inclusion criteria were as follows: 1) patients with pathologically confirmed HNSCC who underwent LNs dissection; 2) age ≥18 years; 3) karnofsky performance status score ≥70; 4) patients with complete data who undergo contrast-enhanced CT and cervical ultrasound within 2 weeks before surgery in our hospital. The exclusion criteria included: 1) patients diagnosed with multiple primary malignant tumors; 2) patients who received any other anti-tumor treatments; 3) patients with a history of liver or kidney dysfunction; 4) patients whose CT or ultrasound presented poor image quality. All patients received tumor extended resection and elective neck dissection. The outcome indicator was cervical LNs metastasis reviewed by two independent pathologists.

### 2.2 Imaging and pathological analysis

A radiotherapy doctor, a radiologist, and a sonographer, each with over 5 years of experience in head and neck cancer imaging, respectively finished their training using randomly selected cases from 10 patients, to reach reasonable agreement in terms of measuring and reporting the imaging findings of the lymph nodes. They meticulously examined and documented the characteristics of the primary tumor, including its size. For metastatic lymph nodes, we gathered information from CT images, including the size of lymph nodes in terms of their long and short axes, the ratio of long to short axes, enhancement patterns, adjacent layer invasion, lymph node aggregation, and the side of lymph node metastasis. On ultrasound images, we assessed factors such as the presence of extracapsular invasion, lymph node echogenicity, hilus and medulla structure and lymph node blood flow of LN. The masses resected by surgery with routine paraffin sectioning and HE-staining were independently reviewed by two pathologists with more than 5 years of experience. Pathologists examine pathological sections of tumor tissues, including the microscopic observation and analysis of the morphological, structural, and histological features of tumor cells, to determine the tumor’s type and degree of differentiation.

CT scans were performed by 64-slice Optima CT660 (GE Healthcare, USA), 256-slice GE Revolution CT (GE Healthcare, USA), 64-slice Siemens Somatom Sensation CT (Siemens Medical Solutions, Forchheim, Germany), or 320-slice multidetector CT (Toshiba Medicals, Tokyo, Japan). Patients underwent the CT scans with slice thicknesses of 5mm and tube voltages of 120kV. Iohexol (350mg/ml) was administered intravenously (1ml/kg) using a flow of 4 ml/s. Ultrasound scans were performed on ultrasound scanners Philips Epiq 7, Philips iU22 or Philips EnVisor with 7 to 12 MHz linear probe.

### 2.3 Cohort definition and variables

These 272 patients were randomly divided into the training and validation cohorts in a 3:1 ratio. Based on the 8th AJCC, we collected the following potential predictors as study variables, which include baseline demographics at diagnosis (sex, age, height, weight, BMI), tumor features (type, size, degree of differentiation), radiological features of LNs (diameters of long and short axis, long-to-short axis ratio (L/S ratio), enhancement pattern, adjacent planes with infiltration, aggregation of LNs, unilateral or bilateral LNs), features in ultrasound images features(extranodal extension, echogenicity, hilus and medulla of LN, blood flow of LNs). LNs aggregation was defined as presence or clustering of more than three LNs in succession. Infiltration of adjacent plane was defined as indistinct LNs’ margins or perinodal soft tissue infiltration.

### 2.4 Statistical analysis

Statistical analysis of the data was performed using SPSS 26.0 software and R software (version 4.1.2, https://www.r-project.org/). First, the caret package was used to randomly divide the patients into the training cohort and validation cohort conforming to the ratio of 3:1. Second, SPSS was used to analyze the correlation and difference of different factors in the training cohort and validation cohort. The Mann–Whitney U test was used to compare continuous data, categorical data were tested by the chi-square test or Fisher’s exact test, Third, multivariate logistic regression models were developed to study effects of predictor variables. For the construction of the multivariable model, we used a forward model building approach. Fourth, we built a nomogram model by using the nomogram package. The nomogram was drawn step-by-step according to the method of Zhang et al (6). Subsequently, Model’s performance was evaluated by AUC and C-index using survival package and pROC package, and we also assessed discrimination, calibration, and clinical utility value by calibration curves and clinical decision curves using lrm package and rmda package. Two-sided *P* values < 0.05 were considered statistically significant.

## 3 Results

### 3.1 Clinical characteristics

**Figure 1:**
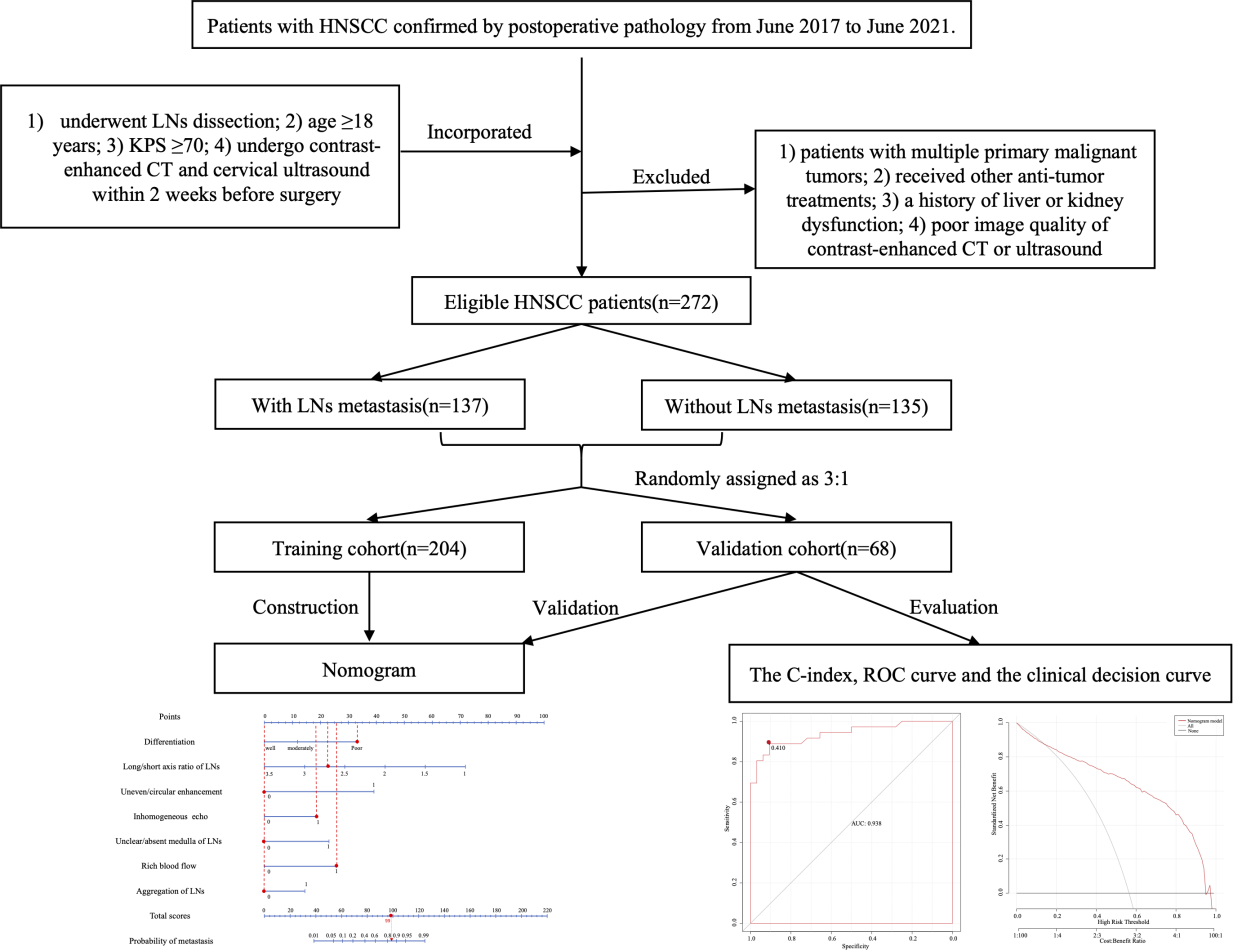
Schematic flow diagram for the process of our study. A total of 272 patients were included, 137 patients of them were cervical LNs positive, while 135 patients were cervical LNs negative. All patients were randomly divided into the training and validation cohorts in a 3:1 ratio, and after screening risk factors by logistic regression, nomogram was developed for predicting lymph nodes metastasis, then the prediction model was verified by C-index, ROC curve, and calibration curve. HNSCC, head and neck squamous cell carcinoma; KPS, karnofsky performance status score; CT, computed tomography; LN, lymph node; ROC, receiver operating characteristic.

The study was conducted as shown in the low chart (**Figure 1**). A total of 272 patients who met the criteria [mean age (SD), 62.99(4.89) years; 256 males and 16 females] were included in this study and their clinical, medical imaging and pathological data were collected for analysis. According to the postoperative pathological results of cervical LNs, the patients were divided into the two following groups: the nonmetastatic group (*n*=135) and the metastatic group (*n*=137). Comparing the data of the two groups (**Table 1**). There was a significant difference between the two groups in age (64.27 ± 9.04 vs. 62.20 ± 9.15, *P*=0.048), and no significant difference in sex(*P*=0.053), BMI(*P*=0.408), height(*P*=0.718), weight(*P*=0.396), and tumor type (*P*=0.113).

**Table 1:** Baseline demographics and tumor type of the HNSCC patients.

Variables	Non-metastasis	Metastasis	*p*
	(*n*=135)	(*n*=137)	
**Age(year)**	64.27 ± 9.04	62.20 ± 9.15	0.048*
**Gender**			0.530
Female	9	7	
Male	126	130	
**BMI(kg/m2)**	23.04 ± 2.79	22.82 ± 3.40	0.408
**Height(cm)**	169.27 ± 2.39	169.46 ± 6.76	0.718
**Weight(kg)**	62.22 ± 10.20	65.75 ± 12.01	0.396
**Tumor type**			0.113
Larynx carcinoma	68	64	
Oral carcinoma	23	23	
Oral floor carcinoma	10	3	
Hypopharyngeal carcinoma	34	47	

Data are presented as mean ± standard deviation or n. *, statistical significance (P<0.05).

### 3.2 Grouping and comparison of the training and validation cohorts

The 272 patients were randomly divided into the training cohort(n=204) and validation cohort(n=68), and no significant differences were found between the two groups (*P* > 0.05), indicating the consistency of these two groups (**Table 2**).

**Table 2:** Comparison of the training and validation cohorts.

Variables	Training cohort	Validation cohort	*P*
	(*n*=204)	(*n*=68)	
**Group**			0.73
Non-metastatic group	103	32	
Metastatic group	101	36	
**Age(year)**	63.38 ± 8.58	62.78 ± 10.67	0.54
**Gender**			0.77
Female	13	3	
Male	191	65	
**BMI (kg/m2)**	22.79 ± 3.09	22.37 ± 3.14	0.13
**Height(cm)**	169.09 ± 6.37	170.18 ± 7.12	0.19
**Weight (Kg)**	62.22 ± 10.12	67.96 ± 11.84	0.07
**Tumor type**			0.34
Larynx carcinoma	101	31	
Oral carcinoma	34	12	
Oral floor carcinoma	7	6	
Hypopharyngeal carcinoma	62	19	
**Diameter of long axis(mm)**	12.51 ± 8.28	14.95 ± 9.62	0.08
**Diameter of short axis (mm)**	8.00 ± 6.25	9.77 ± 7.39	0.08
**L/S ratio**	1.76 ± 0.48	1.71 ± 0.43	0.61
**Adjacent plane with infiltration**			0.14
With	9	4	
Without	195	64	
**Aggregation of LNs**			0.12
With	75	33	
Without	129	35	
**Enhancement pattern**			0.84
Uneven/circular	36	12	
Obvious	6	3	
Mild/without	162	53	
**Side**			0.75
Unilateral	150	48	
Bilateral	54	20	
**Capsule**			0.55
Clear/sharp	164	52	
Extension	40	16	
**Medulla of LNs**			0.39
Clear	128	38	
Unclear/absent	76	30	
**Hilus of LNs**			0.40
Clear	150	46	
Unclear/absent	54	22	
**Blood flow**			0.24
Rich	171	52	
Without	33	16	
**Echogenicity**			0.75
Homogeneous	161	48	
Inhomogeneous	43	20	
**Differentiation degree**			0.96
Well	54	19	
Moderately	121	39	
Poor	29	10	
**Longitudinal diameter of tumor(cm)**	2.71 ± 1.19	2.87 ± 1.44	0.83

Data are presented as mean ± standard deviation or n.

Furthermore, patients in the training cohort were divided into the non-metastatic group (n=103) and the metastatic group (n=101) according to pathology. Comparing the data of these two groups, the following variables as follows showed statistically significant differences between these two groups (P < 0.001): the diameters of the long and short axis of the LNs, the L/S ratio, adjacent planes with infiltration, aggregation of LNs, enhancement pattern, extranodal extension, ultrasound echogenicity, hilus and medulla of LNs, blood flow, differentiation degree and longitudinal diameter of primary tumor (**Table 3**).

**Table 3:** Statistical analysis of the training and validation cohorts.

Variables	Training cohort			Validation cohort		
	Non-metastatic group(*n*=103)	Metastatic group(*n*=101)	*P*	Non-metastatic group(*n*=32)	Metastatic group (*n*=36)	*P*
**Age**	64.39 ± 8.55	62.46 ± 8.57	0.058	63.90 ± 10.58	61.78 ± 10.80	0.491
**Gender**			0.890			0.598
Female	7	6		2	1	
Male	96	95		30	35	
**BMI (kg/m2)**	22.79 ± 2.76	22.77 ± 3.41	0.784	23.83 ± 2.79	22.95 ± 3.40	0.221
**Height(cm)**	169.38 ± 6.13	168.80 ± 6.62	0.685	168.91 ± 7.75	171.31 ± 6.91	0.193
**Weight (Kg)**	62.22 ± 10.12	169.09 ± 6.37	0.416	68.23 ± 10.45	67.72 ± 13.10	0.676
**Diameter of LNs**			<0.001*			<0.001*
Diameter of long axis(mm)	8.44 ± 4.84	16.65 ± 9.01		10.01 ± 5.78	19.32 ± 10.28	
Diameter of short axis (mm)	4.57 ± 3.32	11.50 ± 6.60		5.27 ± 3.48	13.76 ± 7.67	
L/S ratio	1.98 ± 0.36	1.53 ± 0.48		1.99 ± 0.28	1.46 ± 0.38	
**Adjacent planes of infiltration**			0.003*			0.616
With	1	8		1	3	
Without	102	93		31	33	
**Aggregation of LNs**			<0.001*			0.009*
With	23	52		10	23	
Without	80	49		22	13	
**Enhancement**			<0.001*			<0.001*
Uneven/circular	2	34		0	12	
Obvious	1	5		1	2	
Mild/without	100	62		31	22	
**Side**			0.342			0.287
Unilateral	79	71		25	23	
Bilateral	24	30		7	13	
**Capsule**			<0.001*			<0.001*
Complete	96	68		31	21	
Extension	7	33		1	15	
**Medulla**			0.001*			<0.001*
Clear	91	37		27	11	
Unclear/absent	12	64		5	25	
**Hilus of LNs**			0.002*			0.002*
Clear	94	56		28	18	
Unclear/absent	9	45		4	18	
**Blood flow**			0.004*			0.002*
Rich	2	31		2	14	
Poor	101	70		30	22	
**Ultrasound echo**			<0.001*			0.001*
Homogeneous	97	64		29	29	
Inhomogeneous	6	37		3	3	
**Differentiation**			<0.001*			0.383
Well	41	41		11	8	
Moderately	55	55		18	21	
Poor	7	7		3	7	
**Diameter of tumor(cm)**	2.46 ± 1.09	2.95 ± 1.24	0.002*	2.40 ± 1.02	3.29 ± 1.62	0.012*

Data are presented as mean ± standard deviation or n. *, statistical significance (P<0.05).

### 3.3 Single- and multifactor regression analysis

Differentiation degree and diameter of primary tumor, L/S ratio of LNs, enhancement pattern, aggregation of LNs, unilateral/bilateral, ultrasound echogenicity, hilus and medulla of LNs, blood flow and extranodal extension were included in the single regression analysis (**Table 4**). In the training cohort, unilateral/bilateral [95% confidence interval (CI): 0.744~2.599, *P*=0.301] was not identified as a risk factor for LNs metastasis, and the differences in other variables were statistically significant. We included them in the multifactorial regression model (**Table 4**), and the results showed that the differentiation degree of the tumor (OR:2.871, 95%CI: 1.224~6.735, *P*=0.015), L/S ratio of LNs (OR: 0.064, 95%CI:0.019~0.217, *P*<0.001), inhomogeneous enhancement (OR: 4.966, 95%CI: 1.476~16.715, *P*=0.010), LNs aggregation (OR: 3.860, 95%CI: 1.373~10.849, *P*=0.010), inhomogeneous echo (OR: 5.591, 95%CI: 1.337~23.389, *P*=0.018), rich blood flow (OR: 12.927, 95%CI: 1.952~85.632, *P*=0.008), and unclear/absent medulla (OR: 10.516, 95%CI: 2.514~43.989, *P*=0.001) were risk factors for cervical LNs metastasis.

**Table 4:** Univariate and multivariate analysis of the training cohort.

Variables	Univariate		Multivariate	
	OR (95%CI)	*P*	OR (95%CI)	*P*
**Differentiation**	3.283 (1.962~5.495)	<0.001*	2.871 (1.224~6.735)	0.015*
**L/S ratio**	0.081 (0.036~0.185)	<0.001*	0.064 (0.019~0.217)	<0.001*
**Uneven/circular enhancement**	7.518 (2.971~19.024)	<0.001*	4.966 (1.476~16.715)	0.010*
**Aggregation of LNs**	3.691 (2.014~6.767)	<0.001*	3.860 (1.373~10.849)	0.010*
**Unilateral/bilateral**	1.391 (0.744~2.599)	0.301		
**Diameter of long axis**	1.440 (1.122~1.849)	0.004*	1.275 (0.805~2.019)	0.301
**Inhomogeneous echo**	9.346 (3.730~23.418)	<0.001*	5.591 (1.337~23.389)	0.018*
**Unclear/absent medulla**	13.117 (6.351~27.094)	<0.001*	10.516 (2.514~43.989)	0.001*
**Unclear/absent hilus**	8.206 (3.726~18.076)	<0.001*	0.943 (0.182~4.892)	0.945
**Rich blood flow**	22.143 (5.131~95.553)	<0.001*	12.927 (1.952~85.632)	0.008*
**Capsule extension**	7.963 (3.161~20.059)	<0.001*	1.367 (0.271~6.900)	0.705

*, statistical significance (P<0.05).

### 3.4 Nomogram development and assessment of consistency, calibration, and discrimination

**Figure 2:**
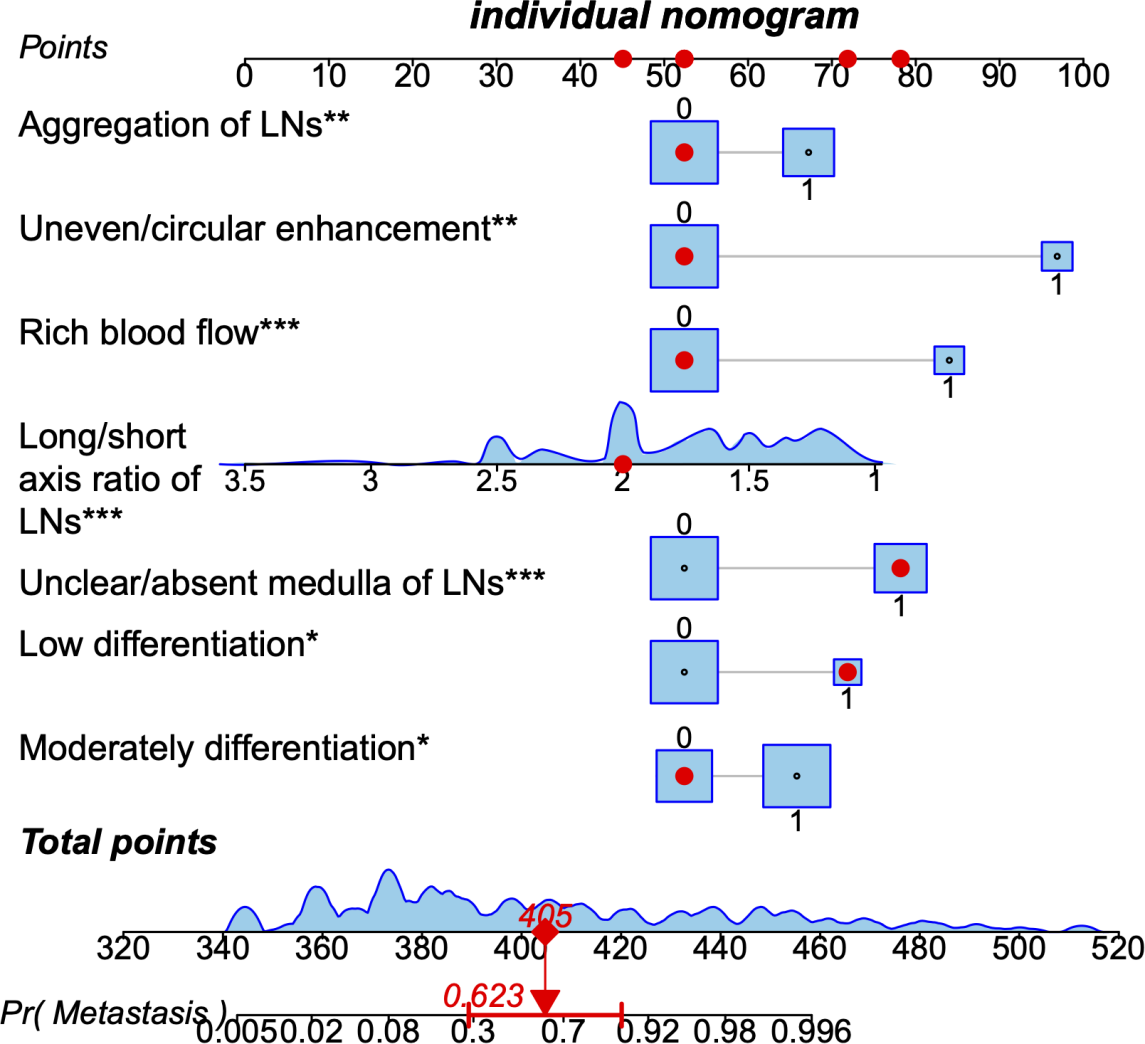
A nomogram predicting the probability of metastasis in cervical lymph nodes in patients with head and neck squamous cell carcinoma. The application of the nomogram is shown by a representative patient. Red points at each scale indicate represented the value of seven predictors. Based on the total value of the seven predictors, the probability of cervical lymph nodes metastasis is about 0.85. LNs, lymph nodes. * means p≤0.05,**means p≤0.01 , *** means p≤0.001.

**Figure 3:**
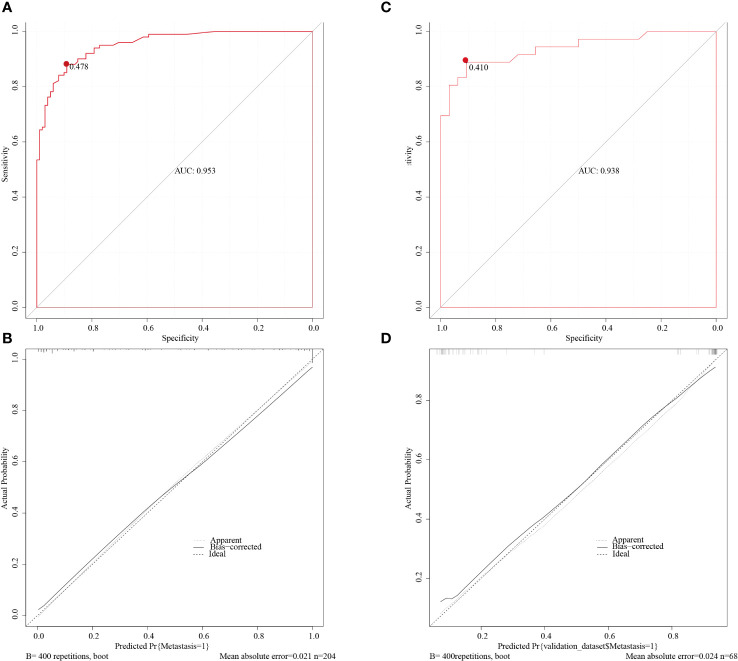
The ROC curve of the training cohort **(A)** and of the validation cohort **(C)**. The AUCs are 0.953 and 0.938 in the training and validation cohorts respectively. The calibration curve of the training cohort **(B)** and of the validation cohort **(D)** indicated good agreement between the predicted probabilities and the actual results in both cohorts. ROC, receiver operating characteristic; AUC, area under curve.

We constructed a nomogram according to the variables screened. The total score could be obtained by summing the individual scores based on the nomogram which corresponds to the probability of cervical LNs metastasis in HNSCC patients (**Figure 2**), it also showed an example of using this to predict the probability of cervical LNs metastasis. The receiver operating characteristic (ROC) curve was plotted with area under curve (AUC) of 0.953 and a cut-off value of 0.478 was determined to differentiate between high and low risks of cervical LNs metastasis (**Figure 3A**). Risk stratification according to the nomogram shows that patients with a score > 80 are high-risk groups for cervical lymph node metastases, a score ≤ 80 are the low-risk groups for cervical lymph node metastasis. Corrected with the calibration curve (**Figure 3B**), the mean absolute error between the predicted and actual values was 0.021, which showed a good degree of compliance.

### 3.5 Validation cohort for evaluating model differentiation

**Figure 4:**
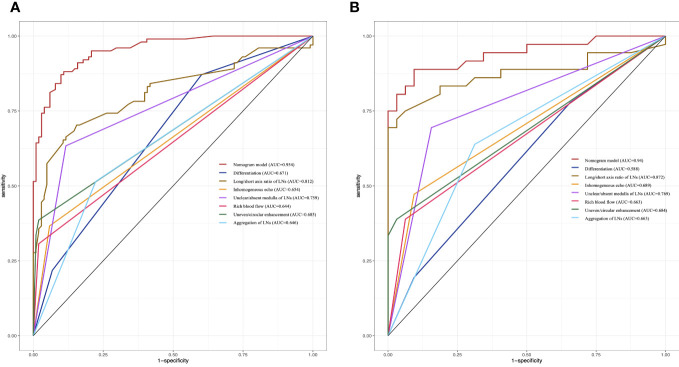
Predictive ROC analysis of individual metrics in the nomogram for lymph node metastasis of training cohort **(A)** and validation cohort **(B)**.

**Figure 5:**
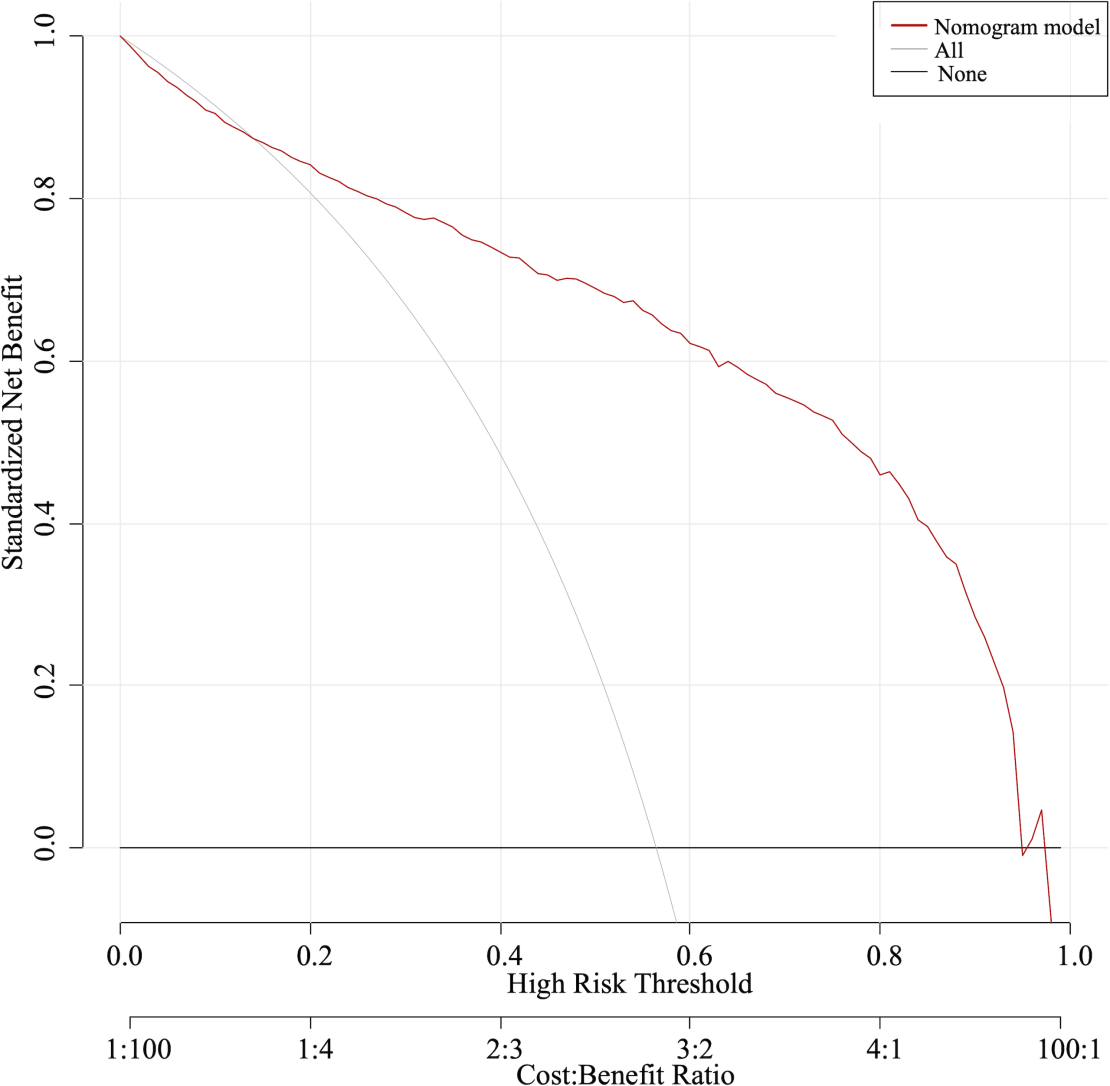
The clinical decision curve indicated a net benefit between 0.25 and 0.9 for the probability of cervical LNs metastasis.

The nomogram was validated with the validation cohort, and the ROC curve was plotted with an AUC of 0.938 (**Figure 3C**). Then, corrected with the calibration plot (**Figure 3D**), the mean absolute error between the predicted and actual values was 0.024. Predictive ROC analysis of individual metrics in the nomogram for lymph node metastasis is shown in **Figure 4**. The predicted risk was close to the actual risk, indicating good model calibration. After 400 bootstrap self-sampling internal tests, the C-index of the model was 0.910, indicating a good agreement between the predicted and the actual situation. The clinical decision curve was plotted (**Figure 5**). The nomogram predicted a net benefit for decision-making across a range of threshold probabilities between 0.25 and 0.9 for the probability of cervical LNs metastasis, with a good performance.

## 4 Discussion

HNSCC is the sixth most common tumor worldwide which is prone to LNs metastasis, there is a significant correlation between cervical LNs metastasis and poor prognosis (7, 8). Typical therapies for HNSCC are surgery and chemoradiotherapy, which have serious side effects. Surgery may cause significant tissue distortion and dysfunction. Chemoradiotherapy may also cause severe dysfunction, such as oral dryness, tissue necrosis, atrophy or fibrosis of bones and soft tissues, resulting in difficulty swallowing (9). Currently, the treatment modalities depends on the staging, zonation of the LNs and involvement of surrounding structures (10). Even in small aggressive primary tumors, there may be occult metastases in the cervical LNs (11). Therefore, we incorporated imaging features of the LNs and clinical characteristics of patients to construct a model to accurately predict LNs staging, which has reference value for clinicians in making neck management decision before initiating a definitive treatment.

Imaging can more accurately identify asymptomatic metastatic LNs and provide a morphologic assessment of such LNs, thus, it’s becoming an effective diagnostic tool for determining treatment strategies (12). Ultrasound and CT are the traditional imaging techniques commonly used for the clinical detection of cervical LNs, because they are easily accessible and non-invasive, but both also have limitations. CT is the often first-choice imaging technique for evaluating cervical LNs and as an objective technique, it’s less subjective. In addition, the use of standard iodine contrast in contrast-enhanced CT scans allows a more accurate evaluation of tumor morphology, including size, contour, margins, and internal structure. Ultrasound is a valuable diagnostic tool in tumor staging and follow-up. The advantages of ultrasound include wide availability, good tolerability, and no radiation exposure, with the added advantage of detecting tumor invasion of blood vessels, and can be used in conjunction with fine-needle aspiration cytology (FNAC) examination (13, 14). Studies have shown that ultrasound provides information on LNs’ location, medullary features, internal echoes, and blood flow distribution. Furthermore, it can be repeated but more subjective than CT scanning (15). Based on the advantages and disadvantages of these two imaging techniques, our study evaluated the accuracy of contrast-enhanced CT and ultrasound in diagnosing cervical LNs metastases in patients with HNSCC. A recent meta-analysis showed that the sensitivity of CT for diagnosing cervical LNs was 77-81% and ultrasound was 76-93% (16). The difference in diagnostic efficacy between the two methods was not statistically significant, neither could be used to accurately detect occult cervical LNs metastasis due to the low negative predictive value of both. Our findings are consistent with previously published results, the sensitivity of neither CT nor ultrasound is 100%, the rate of false-negative results of both methods is relatively high: 21.9% for CT and 17.5% for ultrasound, suggesting that they are not sufficiently reliable methods to exclude the presence of metastatic cervical LNs. So, in these patients especially in stage T1 and T2 tumors, we cannot decide not to perform elective neck dissection based on a “negative” preoperative staging result and should still follow treatment guidelines in making treatment decisions.

Multivariate analysis can obtain coefficients for risk factors and calculate specific risk values through model formulas, but it is difficult to incorporate the predicted values of these indicators (17, 18). Researchers are increasingly interested in nomogram, which is an intuitive graph based on logistic regression results (19). For clinical applications, one of the main advantages of nomograms is the ability to estimate individualized risks based on patients’ and disease’s characteristics. Our study constructed and validated a prediction model based on imaging and pathological parameters for predicting the probability of metastasis in cervical LNs in patients with HNSCC. Poorly differentiated tumors are widely considered to be highly malignant, and previous studies have shown that tumor differentiation is an important prognostic factor for HNSCC and an independent predictor of cervical LNs metastasis (20–22). Consistent with the results of previous studies, our findings suggest that the degree of tumor differentiation is significantly associated with the risk of cervical LNs metastasis. The number, size and anatomical location of LNs were the only criteria used in the clinical staging system (23, 24). LNs’ size is the most discussed criterion for assessing cervical LNs metastasis, and there is a wide variation in the size of reactive and metastatic LNs; however, the challenge is the trade-off between sensitivity and specificity, which increases with decreasing size. Although various criteria have been proposed over the past few decades, however, none is perfect (25).

Therefore, investigators incorporated LNs shape into the imaging criteria to improve the sensitivity of diagnosis (26). Normal and reactive LNs tend to be oval or reniform. In contrast, metastatic LNs are round or spherical. The L/S ratio of LNs has been suggested to quantify LNs shape and predict LNs metastasis (27). Based on these studies, we evaluated the L/S ratio on CT images, it showed that the ratio was negatively correlated with cervical LNs metastasis. LNs aggregation was defined as three or more adjacent LNs. The combination of LNs aggregation and size criteria can improve the sensitivity of the diagnosis without compromising specificity. In our study, LNs aggregation was an independent risk factor for cervical LNs metastasis. In addition, abnormalities in the internal structure of LNs are the most reliable imaging finding in patients with HNSCC, regardless of node size (28, 29). When the tumor spreads to the cervical LNs, the internal structure of the LNs is disrupted, and the tumor cell areas, keratin pooling areas, necrotic areas and residual normal LNs tissues are diffusely distributed. Based on the altered internal structure, the heterogeneity of metastatic LNs leads to an inhomogeneous enhancement or circular enhancement on CT imaging, and inhomogeneous echo, loss of hilus and medulla on ultrasonic imaging (30, 31). Our results also showed the non-homogeneity of metastatic LNs in both CT and ultrasound imaging features. Furthermore, although studies have shown that the loss of hilus is considered as the diagnostic criteria for metastatic LNs on ultrasound (32), however, in the early stages of tumors, the hilus of LNs can still be observed because they have not been sufficiently disrupted (31). In our study, the absence of hilus differed between these two groups but not an independent risk factor for LNs metastasis, we speculate that it is because of the larger proportion of patients with T2 stage in our study population. Normal and reactive LNs usually show either a lymphatic portal vascular distribution or no vascular distribution. Tumors can infiltrate LNs and produce tumor angiogenic factors, leading to angiogenesis and peripheral vascular recruitment, lymphatic portal vessels are preserved until they are destroyed by tumor cells later, so mixed vascular distribution is seen in malignant LNs (33). Ultrasound assessment of the vascular pattern of cervical LNs has been reported to be highly reliable, with a reproducibility of 85% (34). In this study, blood flow characteristics of cervical LNs were evaluated, and the presence of irregular or abundant blood flow in LNs is a valid indicator of LNs metastasis. Individual imaging parameters may be nonspecific, but when appropriately combined and applied, they can improve the accuracy of LNs disease assessment. Our study combined imaging features of ultrasound and postcontrast CT with the degree of pathological tumor differentiation, making the nomogram become more accurate for LNs risk stratification. In conclusion, we believe that this nomogram to predict the risk of LNs metastasis has a theoretical and clinical basis, also can provide reference value and auxiliary diagnosis for clinicians in making neck management decisions of HNSCC patients.

Our study also has some limitations. The size of the tumor has been described in the literature as one of the most important factors affecting LNs metastasis (35). In this study, we observed that tumor length was not an independent risk factor for cervical LNs metastasis, which may be related to the high number of patients with early stage in our case. Early-stage tumors, due to their smaller size, may lack the statistical power to demonstrate independence in relation to LN metastasis. Thus, our conclusions might be limited by a selection bias towards early-stage cases. Future research should include a more diverse range of tumor stages to assess the relationship more accurately between tumor size and LN metastasis. In addition, in both CT and ultrasound imaging parameters, we did not find the LNs border is an independent risk factor for predicting cervical LNs metastasis, but earlier reports showed that metastatic LNs tend to have well-defined sharp borders. In contrast, benign LNs usually show indistinct borders (36). Such sharp borders are attributed to increased acoustic impedance differences between the metastatic node and the surrounding tissue due to tumor infiltration (31). Advanced metastatic LNs may exhibit ill-defined borders, indicating the presence of extranodal extension (ENE). Studies have shown that ENE, a prognostic factor for HNSCC, significantly reduces the 5-year survival of patients and is associated with higher rates of local recurrence and distant metastasis (37). Thus, we should expand the sample size to analyze the role of ENE in LNs metastasis.

Due to limited research resources, this study was a small-scale single-center retrospective study, selection bias and subjective differences in the review of images by ultrasound physicians and radiologists could not be completely excluded, the external validity of their results may be limited. While our study provides valuable insights and contributes to the existing body of knowledge, we acknowledge that its single-center nature constitutes a limitation in terms of broader applicability. Future multicenter studies are recommended to validate our findings across diverse populations and healthcare settings, thus enhancing the external validity and impact of the research. Moreover, we only performed a self-test with 400 resamples of the same population and validated data from the same institution, which may lead to overfitting of the model, so independent data should be selected for external validation at other institutions. Validation of multicenter or even prospective data with large sample sizes would better improve the predictive efficacy of this nomogram and provide a higher level of evidence for clinical application.

## 5 Conclusion

We established and verified the prediction model for cervical lymph node metastasis in HNSCC patients with good performance and can provide auxiliary diagnosis for clinicians.

## Data availability statement

The raw data supporting the conclusions of this article will be made available by the authors, without undue reservation.

## Ethics statement

The studies involving humans were approved by Ethics Committee of the affiliated hospital of Qingdao University. The studies were conducted in accordance with the local legislation and institutional requirements. Written informed consent for participation was not required from the participants or the participants’ legal guardians/next of kin in accordance with the national legislation and institutional requirements.

## Author contributions

XHC and LZ contributed to conception and design of the study. XHC and LZ organized the database. XHC performed the statistical analysis. LZ wrote the first draft of the manuscript. YT, BL and HJL wrote sections of the manuscript. All authors contributed to manuscript revision, read, and approved the submitted version.
